# The modified massive cuff stitch: functional and structural outcome in massive cuff tears

**DOI:** 10.1186/1749-799X-8-26

**Published:** 2013-08-07

**Authors:** Masafumi Gotoh, Yasuhiro Mitsui, Kazuhiro Yoshimitsu, Kenjiro Nakama, Takahiro Okawa, Fujio Higuchi, Kensei Nagata

**Affiliations:** 1 Department of Orthopaedic Surgery, Kurume University Medical Center Kurume, 155-1 Kokubu-machi, Kurume, Fukuoka 839-0863, Japan; 2 Department of Orthopaedic Surgery, Kurume University, 67 Asahi-machi, Kurume, Fukuoka 830-0011, Japan

**Keywords:** Massive cuff tear, Massive cuff stitch, Transosseous suture, Magnetic resonance imaging, Post-operative re-tear

## Abstract

**Background:**

The massive cuff stitch (MCS) is known to be a strong suture, suitable for rotator cuff repair. We modified this technique for massive cuff tears by employing a horizontal medial mattress suture from an anchor as well as a vertically crossing transosseous suture.

**Methods:**

We included 42 patients with massive cuff tears suitable for repair: 22 were treated with the modified MCS (MCS group), and 20 with a simple transosseous suture (STS group). The range of motion (ROM), muscle strength, visual analog scale, and the Japanese Orthopaedic Association (JOA) scores were evaluated pre-operatively and 12 and 24 months post-operatively. The incidence of post-operative re-tears was examined at least 1 year post-operatively using Sugaya's classification.

**Results:**

The ROM, muscle strength, degree of pain, and the JOA scores were much improved after surgery in both groups, and there was no significant intergroup difference throughout the pre- and post-operative periods. In contrast, post-operative MRI revealed a significantly lower re-tear rate in the MCS group than in the STS group (9.1% vs*.* 40%, *P* = 0.0296).

**Conclusions:**

The techniques tested were comparable in terms of functional outcome after surgical repair of massive cuff tears; however, the modified MCS repair technique produced superior structural outcomes with a significantly lower re-tear rate.

## Introduction

Surgical intervention for rotator cuff tears using both arthroscopic and open techniques has resulted in good outcomes [1-4]. Successful healing after rotator cuff repair is dependent on maintenance of the tendon-to-bone reattachment. Failure of the cuff repair may occur at the bone fixation site, the suture, or the tendon. The size of the rotator cuff tear is known to be an important factor influencing the structural outcome after surgical repair [2,4-7]. Repairs for massive rotator cuff tears are technically difficult and are associated with a higher re-tear rate [2,4-6]. Therefore, strong sutures are essential for preventing re-tears after surgical repair of massive cuff tears.

Several studies have shown that hybrid double-row repairs (suture anchor + transosseous suture) are stronger than repairs performed using either a suture anchor or transosseous tunnel alone, as the tendon-to-bone attachment area is greater [8]. The massive cuff stitch (MCS) combines a simple vertical stitch and a horizontal mattress stitch using a single anchor that has a tendon-grasping effect and biomechanical strength equivalent to that of the modified Mason-Allen stitch [3,9,10]. In this context, we were prompted to modify the MCS to take advantage of the tendon-grasping effect and widen the attachment area at the repair site in order to obtain a suitable technique for surgical repair of massive rotator cuff tears. The purpose of the present study was to compare the functional and structural outcomes of the modified MCS and conventional simple transosseous repair techniques. The hypothesis was that the modified MCS repair technique would have a superior structural outcome relative to the simple transosseous repair technique.

## Materials and methods

### Patient selection

A total of 62 patients underwent open surgery between June 2005 and April 2007 for repair of rotator cuff tears. The surgery for the first 30 patients was performed using the simple transosseous suture (STS) repair technique, and the surgery for the remaining 32 cases was performed using the modified MCS technique. The inclusion criteria were as follows: (1) the largest dimension of the ruptured site measured during surgery was >5 cm or the involvement of at least two tendons [4,6], (2) individuals for whom at least 6 months of conservative treatment had failed, and (3) individuals available for pre- and post-operative assessment with the informed consent to participate in this study. Exclusion criteria were as follows: (1) irreparable massive tears, (2) fractures around the shoulder, (3) moderate to marked osteoarthritis of the glenohumeral joint, (4) previous shoulder surgery, and (5) infection.

A total of 42 patients with rotator cuff tears were candidates for this study: 22 were treated with the modified MCS (MCS group), and 20 with STS repairs (STS group). The mean age at the time of surgery was 61.1 ± 6.7 years in the modified MCS group and 61.3 ± 8.4 years in the STS group. The mean time after the onset of symptoms was 3.8 ± 2.8 months in the modified MCS group and 5.1 ± 3.1 months in the STS group. A single surgeon (MG) performed all surgeries. Approval from the Institutional Review Board in our institute was obtained (#13037).

### Data collection

Pre- and post-operative evaluations included a patient questionnaire and a physical examination performed by an independent examiner who was blinded to the study. The examination consisted of functional assessment based on the Japanese Orthopedic Association (JOA) scoring system [11], range of motion testing, and strength testing. A visual analog scale (VAS) was used to rate the patient's perceived level of pain in the range 0–10 [12]. These examinations were performed pre-operatively and at 12 and 24 months post-operatively. These data were obtained from physical therapists blinded to this study.

The patients in the present study underwent magnetic resonance imaging (MRI) (1.5-Tesla MRI unit, Excelart, Toshiba Medical Systems, Tokyo, Japan) at our institution both pre- and post-operatively. The MRI protocol was as described previously [11]. All scans were judged to be adequate for the assessment of rotator cuff tendon integrity. Post-operative re-tears were examined at least 1 year post-operatively (mean, 15.2 months; range, 12–18 months) using Sugaya's classification: type I, repaired cuff with sufficient thickness and homogenous low intensity images; type II, sufficient thickness with a partial high-intensity area; type III, insufficient thickness but without any discontinuity, suggesting a partial-thickness delaminated tear; type IV, presence of a minor discontinuity in only one or two slices on both coronal and sagittal images, suggesting a small full-thickness tear; and type V, the presence of a major discontinuity in more than two slices on both coronal and sagittal images, suggesting a medium or large full-thickness tear [13]. The MR images were read independently by orthopedic specialists and radiologists trained on the musculoskeletal anatomy blinded to the study. A consensus discussion determined the final reading [11].

### Surgical technique

All procedures were performed in beach-chair position. Skin incisions were made along Langer's lines just lateral to the coracoid process. The deltoid was split longitudinally in the raphe, and the insertion of the anterior deltoid was detached from the acromion. After skeletonizing the acromion, an acromioplasty was performed with a chisel. After mobilizing the retracted cuff and excising its margin, the ruptured cuff was sutured. A bone tunnel at the greater tuberosity corresponding to the repair site was made with a chisel. Single interrupted sutures were placed at the lateral edge of the torn cuff and passed through the bone tunnel with 2.4-mm Kirschner wire and large needle (3 cm in diameter).

**Figure 1:**
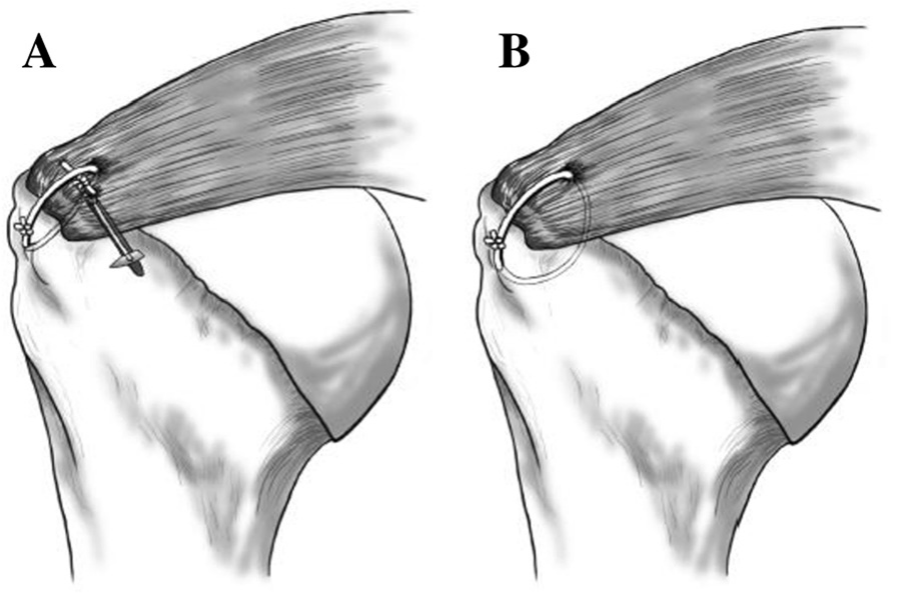
**Schema of the procedure tested.** Modified massive cuff stitch (MCS) **(A)**; simple transosseous suture (STS) **(B)**.

For the modified MCS repair, a horizontal mattress stitch was made on the inside 10–15 mm of the ruptured rotator cuff using a single-loaded bioabsorbable suture anchor (PANALOCK RC, DePuy Mitek Inc., Raynham, MA, USA) placed just medial to the rotator cuff footprint. Next, a vertical simple transosseous suture was made to cross over the horizontal suture proximally after medial tying (Figure 1A). The vertical transosseous suture was then tied. For the STS repair, simple transosseous sutures were placed on the inside 10–15 mm of the ruptured rotator cuff and sutured to the bone tunnel prepared in the greater tuberosity (Figure 1B). Watertight repairs were completed in all patients without excessive tension at the repair site; once repaired, the deltoid was re-attached with non-absorbable braided sutures to its previous anatomical position.

### Rehabilitation after surgery

Elbow, wrist, and finger range of motion exercises were started immediately after surgery. Passive forward elevation of the shoulder commenced the day after surgery. At 4 weeks after surgery, active-assisted motion of the shoulder was initiated, and at 6 weeks, active motion was allowed. Rotator cuff strengthening exercises commenced 10–12 weeks after surgery.

### Statistical analysis

Data are expressed as mean ± SD. A repeated measure analysis of variance was used for comparing pre- and post-operative parameters (JOA score, range of motion, muscle strength, and VAS) in the same procedure. Mann-Whitney *U* test was used for comparing the data from the modified MCS and STS groups. Intergroup differences in the re-tear rate were compared using a chi-squared test. A *P* value < 0.05 was considered significant. All statistical analyses were performed using R software (version 2.15.0, R Foundation for Statistical Computing, Vienna, Austria) [14].

## Results

### Functional outcome

In the MCS repair group, the range of motion in elevation was significantly improved from 95.2° ± 47.1° pre-operatively to 152.1° ± 15.4° at the final follow-up (*P* < 0.05), and the range of motion in abduction from 81.4° ± 44.3° pre-operatively to 144.8° ± 25.4° at final follow-up (*P* < 0.05). The internal rotation was significantly increased from 5.7 ± 2.4 vertebrae pre-operatively to 7.7 ± 2.9 vertebrae at final follow-up (*P* < 0.05). The external rotation was not significantly improved from baseline to final follow-up (41.4° ± 15.7° and 41.7° ± 15.0°, respectively, *P* < 0.05). In the STS repair group, the range of motion was significantly improved from 107.4° ± 43.3° to 149.2° ± 17.3° in elevation (*P* < 0.05) and from 97.1° ± 47.8° to 145.0° ± 27.6° in abduction (*P* < 0.05). There was no significant difference in external and internal rotation from baseline to final follow-up. The details are shown in Table 1.

**Table 1:** Comparison of range of motion between the MCS and STS groups

		**B.O.**	**P.O. 12 M**	**P.O. 24 M**
ELEV	MCS	95.2° ± 47.1°	150.8° ± 15.8°*	152.1° ± 15.4°*
	STS	107.4° ± 43.3°	152.6° ± 13.1°*	149.2° ± 17.3°*
ER	MCS	41.4° ± 15.7°	44.0° ± 15.4°	41.7° ± 15.0°
	STS	42.6° ± 22.7°	41.3° ± 18.8°	39.7° ± 17.8°
IR	MCS	5.7 ± 2.4	7.4 ± 3.1*	7.7 ± 2.9*
	STS	5.9 ± 4.6	7.9 ± 2.8	8.2 ± 3.1
ABD	MCS	81.4° ± 44.3°	139.3° ± 28.6°*	144.8° ± 25.4°*
	STS	97.1° ± 47.8°	151.6° ± 17.3°*	145.0° ± 27.6°*

*ELEV* elevation, *ER* external rotation, *IR* internal rotation, *ABD* abduction, *B.O.* before operation, *P.O.* post-operative, *M* months. *P < 0.05.

Muscle strength was evaluated using a hand-held dynamometer [15] by the same physical therapist throughout the follow-up. In the MCS repair group, when the value was expressed as the ratio of the involved/non-involved side, muscle strength was significantly improved from 56.7 ± 33.8% pre-operatively to 81.2 ± 28.6% in external rotation at final follow-up (*P* < 0.05) and from 79.3 ± 29.9% to 96.2 ± 19.6% in internal rotation at final follow-up (*P* < 0.05). There was no significant improvement in elevation strength or abduction strength from baseline to final follow-up. In the STS repair group, strength in elevation was significantly improved from 55.4 ± 18.3% pre-operatively to 74.2 ± 27.1% (*P* < 0.05), and strength in internal rotation from 85.2 ± 27.2% to 102.8 ± 21.4% at final follow-up (*P* < 0.05). There was no significant improvement in the external rotation strength or abduction strength between baseline and final follow-up. The details are shown in Table 2.

**Table 2:** Comparison of muscle strength between the MCS and STS groups

		**B.O. (%)**	**P.O. 12 M (%)**	**P.O. 24 M (%)**
ELEV	MCS	65.5 ± 26.8	76.3 ± 35.6	78.5 ± 26.1
	STS	55.4 ± 18.3	70.5 ± 19.7*	74.2 ± 27.1*
ER	MCS	56.7 ± 33.8	83.4 ± 27.7*	81.2 ± 28.6*
	STS	70.3 ± 36.2	79.9 ± 26.4	78.4 ± 25.4
IR	MCS	79.3 ± 29.9	95.5 ± 23.7	96.2 ± 19.6*
	STS	82.5 ± 27.2	100.6 ± 11.8*	102.8 ± 21.4*
ABD	MCS	66.7 ± 33.0	76.7 ± 19.7	79.1 ± 23.6
	STS	65.9 ± 24.6	79.9 ± 25.2	79.8 ± 26.6

Values are given as the relative ratio of the involved side to the non-involved side. *ELEV* elevation, *ER* external rotation, *IR* internal rotation, *ABD* abduction, *B.O.* before operation, *P.O.* post-operative, *M* months. *P < 0.05.

The degree of shoulder pain was evaluated by VAS [12]. In the MCS repair group, the degree of pain at rest was significantly improved from 1.7 ± 2.1 pre-operatively to 0.2 ± 1.0 at final follow-up (*P* < 0.05), the degree of pain on motion from 6.3 ± 2.0 pre-operatively to 0.5 ± 1.5 at final follow-up (*P* < 0.05), and the degree of pain at night from 4.0 ± 2.7 pre-operatively to 0.4 ± 1.4 at final follow-up (*P* < 0.05). In the STS repair group, the degree of pain at rest was significantly improved from 2.1 ± 2.6 pre-operatively to 0.4 ± 1.4 at final follow-up (*P* < 0.05), the degree of pain on motion from 5.9 ± 2.9 pre-operatively to 0.9 ± 1.8 at final follow-up (*P* < 0.05), and the degree of pain at night from 5.1 ± 2.8 pre-operatively to 0.7 ± 2.1 at final follow-up (*P* < 0.05). The details are shown in Table 3.

**Table 3:** Comparison of VAS scores between the MCS and STS groups

		**B.O.**	**P.O. 12 M**	**P.O. 24 M**
Static	MCS	1.7 ± 2.1	0.1 ± 0.2*	0.2 ± 1.0*
	STS	2.1 ± 2.6	0.3 ± 0.7*	0.4 ± 1.4*
Dynamic	MCS	6.3 ± 2.0	0.2 ± 0.5*	0.5 ± 1.5*
	STS	5.9 ± 2.9	0.8 ± 1.6*	0.9 ± 1.8*
Night	MCS	4.0 ± 2.7	0.1 ± 0.4*	0.4 ± 1.4*
	STS	5.1 ± 2.8	0.3 ± 1.1*	0.7 ± 2.1*

The degree of VAS shows 10 as severe, 5 as moderate, and 0 as pain free. *B.O.* before operation, *P.O.* post-operative, *M* months. *P < 0.05.

The total JOA score was significantly improved in both groups: from 62.3 ± 9.6 pre-operatively to 91.9 ± 5.5 at final follow-up in the MCS repair group (*P* < 0.05), and from 59.1 ± 7.7 pre-operatively to 89.4 ± 7.6 at final follow-up in the STS repair group (*P* < 0.05). The details are shown in Table 4.

**Table 4:** Comparison of JOA scores between the MCS and STS groups

		**B.O.**	**P.O. 12 M**	**P.O. 24 M**
Total	MCS	62.3 ± 9.6	89.3 ± 5.8*	91.9 ± 5.5*
	STS	59.1 ± 7.7	91.7 ± 4.7*	89.4 ± 7.6*

*B.O.* before operation, *P.O*. post-operative, *M* months. Asterisk shows *P* value less than 0.05.

There was no significant difference in the improvements between the modified MCS and STS repair groups throughout the pre- and post-operative periods.

### Structural outcome

All patients underwent MRI assessment during post-operative year 1 (12–18 months) to determine the integrity of the repaired cuff, by using Sugaya's classification [13]. In the MCS repair group, there were five type-I cases, nine type-II cases, six type-III cases, two type-IV cases, and no type-V cases. In the STS repair group, there was one type-I case, four type-II cases, seven type-III cases, five type-IV cases, and three type-V cases. If type IV and V cases are considered re-tears [13], then the re-tear rate was significantly lower in the MCS repair group (2/22 cases; 9.1%) than that in the STS repair group (8/20 cases; 40%). The details are shown in Table 5.

**Table 5:** **Evaluation of post-operative cuff integrity in the MCS and STS groups according to Sugaya's classification**[13]

	**I**	**II**	**III**	**IV**	**V**
MCS	5	9	6	2	0
STS	1	4	7	5	3

Type IV and type V indicate post-operative tendon re-tears.

## Discussion

Previous studies on massive rotator cuff tears treated by open or arthroscopic surgery have reported a wide range of re-tear rates (40%–94%): 94% by Galatz et al. [2], 40% by Sugaya et al. [16], 33% by Harryman et al. [17], and 34% by Liu and Baker [1]. Recently, Yamaguchi et al. [7] reported that the open suture anchor-augmented repair technique produced a lower re-tear rate (2/24 cases; 8.3%) for massive cuff tears. Our modified MCS also had a relatively low re-tear rate (2/22 cases, 10%).

Apart from the structural outcome, the functional outcome has consistently been satisfactory in patients with large/massive cuff tears: Bigliani et al. [5] reported 85% satisfactory results, and Rokito et al. [18] reported 77% good or excellent results. Yamaguchi et al. [7] reported an average 89.3 JOA score, which corresponds to a 91% satisfactory result if evaluated by UCLA score. Although the present study did not evaluate the UCLA score, we showed that the JOA score averaged 91.9 points and was comparable between the groups.

The MCS was developed to increase the strength of the suture-tendon interface for arthroscopic surgery; it is biomechanically similar to the modified Mason-Allen stitch [10]. Ko et al. showed that for arthroscopic treatment of small/medium rotator cuff tears, the MCS repair had a lower failure rate (14.3%) than the simple suture repair (27.8%) [3]. By modifying this technique and applying it to massive cuff tears, we obtained an acceptable structural outcome with a 10% re-tear rate, which is much lower than the rate obtained with the simple transosseous repair technique (40%).

The original MCS consists of a vertical stitch and a horizontal mattress stitch that originate from the same suture anchor. Pullout of the vertical stitch is prevented by the tendon-grasping effect of the horizontal stitch [19]. The main fixation failure in rotator cuff repair is due to suture pullout from the tendon [2,3,6,10]; therefore, we intended not only to prevent pullout of the vertical loop, but also to increase the bone-tendon contact at the repair site by locating the horizontal medial mattress stitch from the anchor to the vertical transosseous stitch.

Our data also suggest an important role of the medial mattress stitch near the suture anchor for obtaining a good structural outcome. This modified MCS is expected to provide increased bridging compression between the medial stitch and the lateral edge of the vertical transosseous stitch. Thus, our modified MCS may be thought of as a transosseous-equivalent double-row repair, the latter of which has been reported to have greater fixation in rotator cuff repair [20].

The present study had several limitations. First, this study did not perform biomechanical testing of the modified MAC stitch. However, the structural outcome of the present series revealed a favorable outcome compared with previous studies of massive cuff tears [1,2,16,17]. Second, the present study is a retrospective non-randomized cohort study with a small sample size. Third, the STS was done first, and the MCS second; therefore, there could have been learning curve in this case series. Fourth, the MR arthrography to show watertight repair would have been better, thus detecting smaller tears after repair. Actually, there was a different distribution of post-operative cuff integrity between the MCS and STS groups. This could account for the difference in failure as seen on the conventional MRI; however, the previous studies have evaluated post-operative re-tear by this conventional method [7,11,13]. Resolution of these issues will provide further clarification of the outcomes obtained in the present study.

## Conclusions

We compared the clinical outcomes of the modified MCS stitch and the simple transosseous suture in patients with massive cuff tears. We found that the techniques were comparable in terms of functional outcome; however, the modified MCS repair technique produced a superior structural outcome with a significantly lower re-tear rate.

## Competing interests

The authors declare that they have no competing interests.

## Authors’ contributions

The design of the study and preparation of the manuscript were done by MG, YM and KY. KN assisted in the study processes, sample collections, and preparations. TO, FH, and KN assisted in the manuscript preparation. All authors read and approved the final manuscript.
